# Transcriptional effects of ^177^Lu-octreotate therapy using a priming treatment schedule on GOT1 tumor in nude mice

**DOI:** 10.1186/s13550-019-0500-2

**Published:** 2019-03-20

**Authors:** Johan Spetz, Britta Langen, Nils-Petter Rudqvist, Toshima Z. Parris, Emman Shubbar, Johanna Dalmo, Bo Wängberg, Ola Nilsson, Khalil Helou, Eva Forssell-Aronsson

**Affiliations:** 1 Department of Radiation Physics, Institute of Clinical Sciences, Sahlgrenska Cancer Center, Sahlgrenska Academy at University of Gothenburg, Gula Stråket 2B, Sahlgrenska University Hospital, SE-413 45 Gothenburg, Sweden; 2 Department of Applied Physics, Chalmers University of Technology, Gothenburg, Sweden; 3 Department of Oncology, Institute of Clinical Sciences, Sahlgrenska Cancer Center, Sahlgrenska Academy at University of Gothenburg, Gothenburg, Sweden; 4 Department of Medical Physics and Biomedical Engineering, Sahlgrenska University Hospital, Gothenburg, Sweden; 5 Department of Surgery, Institute of Clinical Sciences, Sahlgrenska Cancer Center, Sahlgrenska Academy at University of Gothenburg, Gothenburg, Sweden; 6 Department of Pathology, Institute of Biomedicine, Sahlgrenska Cancer Center, Sahlgrenska Academy at University of Gothenburg, Gothenburg, Sweden

**Keywords:** GEPNET, NET, Radionuclide therapy, Radiation biology, Gene expression, Midgut carcinoid, PRRT, ^177^Lu-DOTATATE

## Abstract

**Background:**

^177^Lu-octreotate is used for therapy of somatostatin receptor expressing neuroendocrine tumors with promising results, although complete tumor remission is rarely seen. Previous studies on nude mice bearing the human small intestine neuroendocrine tumor, GOT1, have shown that a priming injection of ^177^Lu-octreotate 24 h before the main injection of ^177^Lu-octreotate resulted in higher ^177^Lu concentration in tumor, resulting in increased absorbed dose, volume reduction, and time to regrowth. To our knowledge, the cellular effects of a priming treatment schedule have not yet been studied. The aim of this study was to identify transcriptional changes contributing to the enhanced therapeutic response of GOT1 tumors in nude mice to ^177^Lu-octreotate therapy with priming, compared with non-curative monotherapy.

**Results:**

RNA microarray analysis was performed on tumor samples from GOT1-bearing BALB/c nude mice treated with a 5 MBq priming injection of ^177^Lu-octreotate followed by a second injection of 10 MBq of ^177^Lu-octreotate after 24 h and killed after 1, 3, 7, and 41 days after the last injection. Administered activity amounts were chosen to be non-curative, in order to facilitate the study of tumor regression and regrowth. Differentially regulated transcripts (RNA samples from treated vs. untreated animals) were identified (change ≥ 1.5-fold; adjusted *p* value < 0.01) using Nexus Expression 3.0. Analysis of the biological effects of transcriptional regulation was performed using the Gene Ontology database and Ingenuity Pathway Analysis. Transcriptional analysis of the tumors revealed two stages of pathway regulation for the priming schedule (up to 1 week and around 1 month) which differed distinctly from cellular responses observed after monotherapy. Induction of cell cycle arrest and apoptotic pathways (intrinsic and extrinsic) was found at early time points after treatment start, while downregulation of pro-proliferative genes were found at a late time point.

**Conclusions:**

The present study indicates increased cellular stress responses in the tumors treated with a priming treatment schedule compared with those seen after conventional ^177^Lu-octreotate monotherapy, resulting in a more profound initiation of cell cycle arrest followed by apoptosis, as well as effects on PI3K/AKT-signaling and unfolded protein response.

**Electronic supplementary material:**

The online version of this article (10.1186/s13550-019-0500-2) contains supplementary material, which is available to authorized users.

## Background

Neuroendocrine tumors (NETs) have frequently metastasized at the time of diagnosis. Following surgical tumor reduction, adjuvant treatment with ^177^Lu-[DOTA^0^, Tyr^3^]-octreotate (also written as ^177^Lu-octreotate or ^177^Lu-DOTATATE) is used for patients with somatostatin receptor (SSTR)-positive NETs, with complete remission in approximately 2% and partial remission in < 30% of patients [1–3]. ^177^Lu is a medium-energy beta emitter (mean electron energy emitted per nuclear decay 147.9 keV) with a half-life of 6.6 days [4]. The mean range of the beta particles is 0.67 mm, allowing for a relatively contained dose distribution in tumors with high specific uptake of ^177^Lu-octreotate [5].

Several strategies have been proposed to further optimize the therapeutic effect of ^177^Lu-octreotate in NETs, including methods to increase tumor uptake and retention of ^177^Lu-octreotate [6]. We have previously demonstrated that tumor cells with neuroendocrine features increase their expression of *SSTR* after exposure to ionizing radiation in vitro [7, 8]. In vivo studies using the human small intestine NET model, GOT1 xenotransplanted to nude mice [9], have also shown an increased binding of ^111^In-DTPA-octreotide in tumor tissue after injection of ^177^Lu-octreotate [10, 11]. Furthermore, we have also shown a higher concentration of ^177^Lu in tumor tissue after administration of a low amount of ^177^Lu-octreotate (priming dose) given 24 h before the main administration of ^177^Lu-octreotate, compared with that found after single injection of the same total activity [12]. The priming treatment schedule thus resulted in higher mean absorbed dose to the tumor and increased anti-tumor effects. However, radiation-induced upregulation of *SSTR* has not been confirmed in vivo. Therefore, it is necessary to determine the mechanisms involved in the increased treatment efficacy observed when using a priming administration of ^177^Lu-octreotate before a second administration.

We have previously demonstrated the effects of exposure to radionuclides in animal models using expression microarray analysis. Initially, the effects of ^131^I or ^211^At exposure of normal tissues were demonstrated in mice and rats [13–18]. Then, studies on transcriptional effects of ^177^Lu-octreotate exposure of kidneys (to evaluate radiotoxicity) showed different responses in the kidney cortex and medulla [19]. Recently, expression microarray analysis of GOT1 tumors was presented, demonstrating radiation-induced apoptosis as an early response after a non-curative ^177^Lu-octreotate administration, followed by pro-survival transcriptional changes in the tumor during the regrowth phase [20, 21].

The aim of this study was to examine the transcriptional response in tumor tissue from animals treated with a priming administration of ^177^Lu-octreotate 24 h before a second ^177^Lu-octreotate administration to determine the molecular mechanisms responsible for the higher anti-tumor effect in comparison with ^177^Lu-octreotate monotherapy with the same total amount of ^177^Lu-octreotate.

## Methods

### Experimental design

**Fig. 1:**
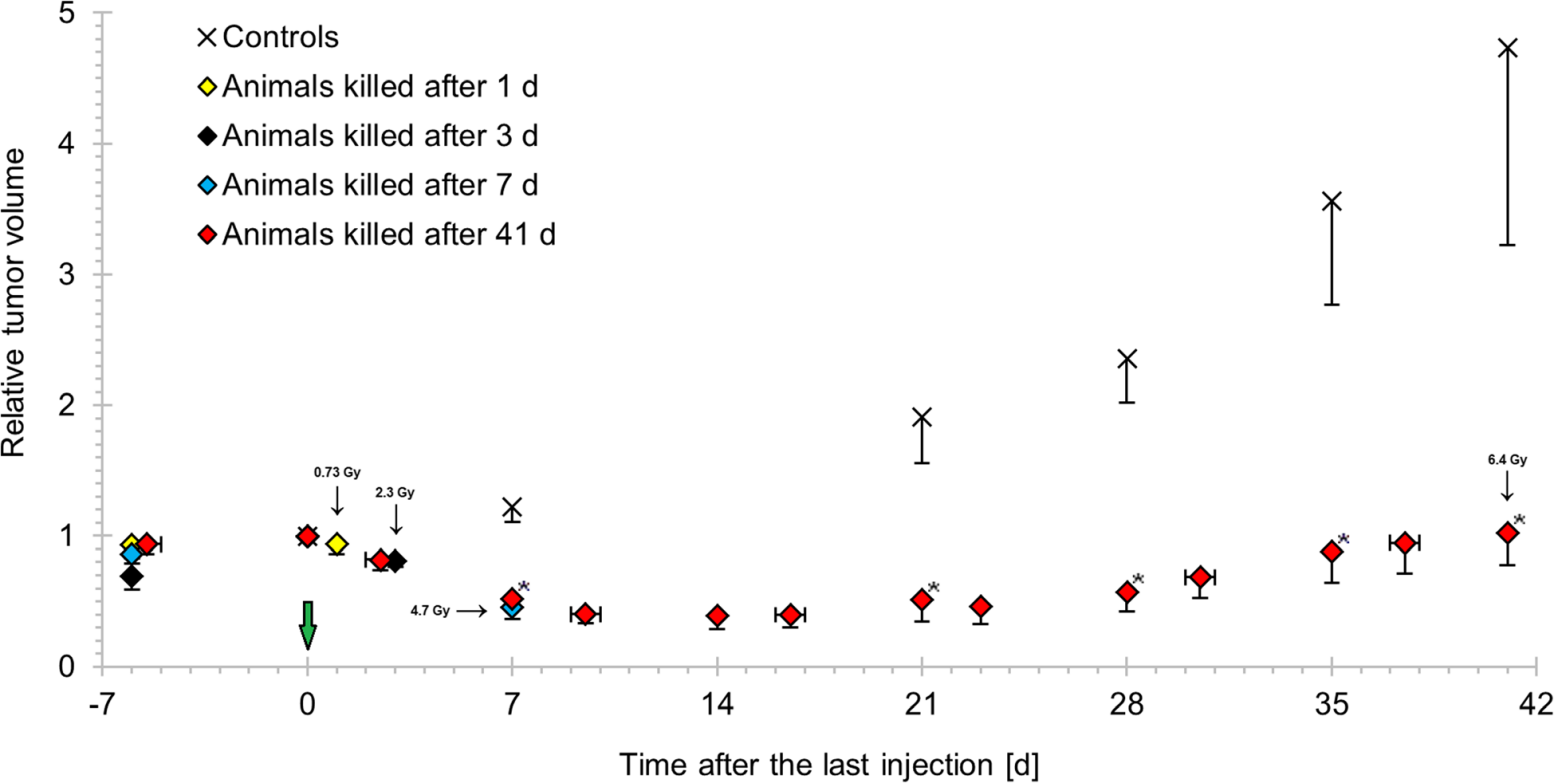
Anti-tumor effect of ^177^Lu-octreotate with priming on GOT1 in nude mice. The mean relative tumor volume versus time after last injection for mice i.v.-injected with 5 + 10 MBq ^177^Lu-octreotate or NaCl and killed at 1, 3, 7 or 41 days after the last injection. The mean absorbed dose to tumors processed for transcriptional analysis is indicated by the corresponding data point. Vertical error bars indicate SEM, and horizontal error bars indicate range. The figure is based on the tumor volume and biodistribution data analyzed in these animals in Dalmo et al. [12]. Green arrow indicates the time for treatment start. Asterisk indicates statistically significant difference versus controls (*t* test, *p* < 0.05)

This study was performed on 24 GOT1 tumor tissue samples obtained from previous experimental studies [12]. Briefly, GOT1 tumor tissue samples were transplanted subcutaneously in the neck of 4-week-old female BALB/c nude mice (Charles River, Japan and Germany) [9]. Tumor-bearing mice received a priming injection of ^177^Lu-octreotate (5 MBq) followed by a second injection of ^177^Lu-octreotate (10 MBq) 24 h later (hereafter referred to as 5 + 10 MBq). Control animals were injected with saline solution. During the study period, tumor volume was monitored using caliper measurement. Mean tumor volume relative to the time of the last injection was reduced in animals treated with ^177^Lu-octreotate (Fig. 1), with statistically significant differences compared with controls from day 7 until end of study (calculated using Student’s *t* test, *p* < 0.05). The minimum relative tumor volume in animals used in this study (mean = 0.39, SEM = 0.11) was measured 14 days after injection in animals killed after 41 days. The animals were killed at 1, 3, 7, or 41 days after the last injection, and tumor samples were frozen in liquid nitrogen and stored at − 80 °C until analysis. Tumor-absorbed doses were determined for the 5 + 10 MBq administrations using the medical internal radiation dose (MIRD) formalism [22]. This resulted in a mean absorbed dose of 0.73, 2.3, 4.7, 6.4, and 6.4 Gy to the tumors calculated to 1, 3, 7, 41 days and at infinite time, respectively (Fig. 1). Drinking water and autoclaved food were provided ad libitum. Gene expression microarray analysis was performed on total RNA extracted from tumor samples from 15 animals treated with 5 + 10 MBq ^177^Lu-octreotate (*n* = 3, 3, 3, and 6 at 1, 3, 7, and 41 days after the second injection, respectively) and nine control animals (*n* = 2, 2, 2, and 3 at 1, 3, 7, and 41 days after injection, respectively).

### Gene expression analysis

RNA extraction, hybridization, and data processing were performed for each of the 24 tumor samples individually as previously described [12]. In brief, total RNA was isolated using the RNeasy Lipid Tissue Mini Kit (Qiagen, Germany). Hybridization of RNA samples (RNA integrity numbers > 6.0) was performed at the Swegene Center for Integrative Biology (SCIBLU, Lund University, Sweden) on Illumina HumanHT-12 v4 Whole-Genome Expression BeadChips (Illumina, USA). Data processing was performed using the BioArray Software Environment (BASE) and Nexus Expression 3.0 (BioDiscovery, USA) [12, 23]. Differentially regulated transcripts (treated versus control) were identified using an adjusted *p* value cutoff of < 0.01 (Benjamini-Hochberg method [24]) and |fold change| ≥ 1.5. The RNA samples from the control animals in this study have previously been used to analyze tumor RNA samples from animals treated with 15 MBq ^177^Lu-octreotate mono-injection, collected at 1, 3, 7, and 41 days after injection [20].

Microarray data were validated using quantitative reverse transcription-polymerase chain reaction (qRT-PCR) performed in triplicate with predesigned TaqMan® assays (Applied Biosystems, USA) specific for *BAX*, *CDKN1A*, *FDFT1*, *GDF15*, *TGFBI*, *ACTA2*, *LY6H*, *LDLR*, and *EGR1* using a 7500 Fast Real-Time PCR System (Applied Biosystems). Differential expression was calculated using the ΔΔCt method, with *EEF1A1*, *RPL6*, and *RPS12* used for normalization. cDNA was synthesized from the same RNA extracted for use in the microarray experiments, using SuperScript™ III First-Strand Synthesis SuperMix (Invitrogen, USA). cDNA reactions without addition of reverse transcriptase prior to qRT-PCR did not monitor any interfering genomic DNA.

### Bioinformatics analysis

Heat maps and unsupervised hierarchical clustering of transcripts based on regulation patterns was performed in the R statistical computing environment (http://www.r-project.org, version 3.5.1), as previously described [20]. Functional annotation of differentially regulated transcripts was performed using the Gene Ontology (GO) database (http://www.geneontology.org) [11], with a *p* value cutoff of < 0.05 (modified Fisher’s exact test). The annotated biological processes were stratified into eight categories as previously described [15]. Analysis of affected biological functions, canonical pathways, and upstream regulators was conducted using the Ingenuity Pathway Analysis (IPA) software (Ingenuity Systems, USA) with Fisher’s exact test (*p* < 0.05) as previously described [20, 21]. For direct comparison of the gene expression data obtained in this study with that of a more conventional treatment schedule (15 MBq single administration of ^177^Lu-octreotate, tumor samples collected at 1, 3, 7, and 41 days after injection), data from National Center for Biotechnology Information (NCBI) Gene Expression Omnibus (GEO), accession GSE80024 (previously described in [20]), was used.

## Results

### Time-dependent transcriptional response in GOT1 tumors after ^177^Lu-octreotate therapy with priming

**Fig. 2:**
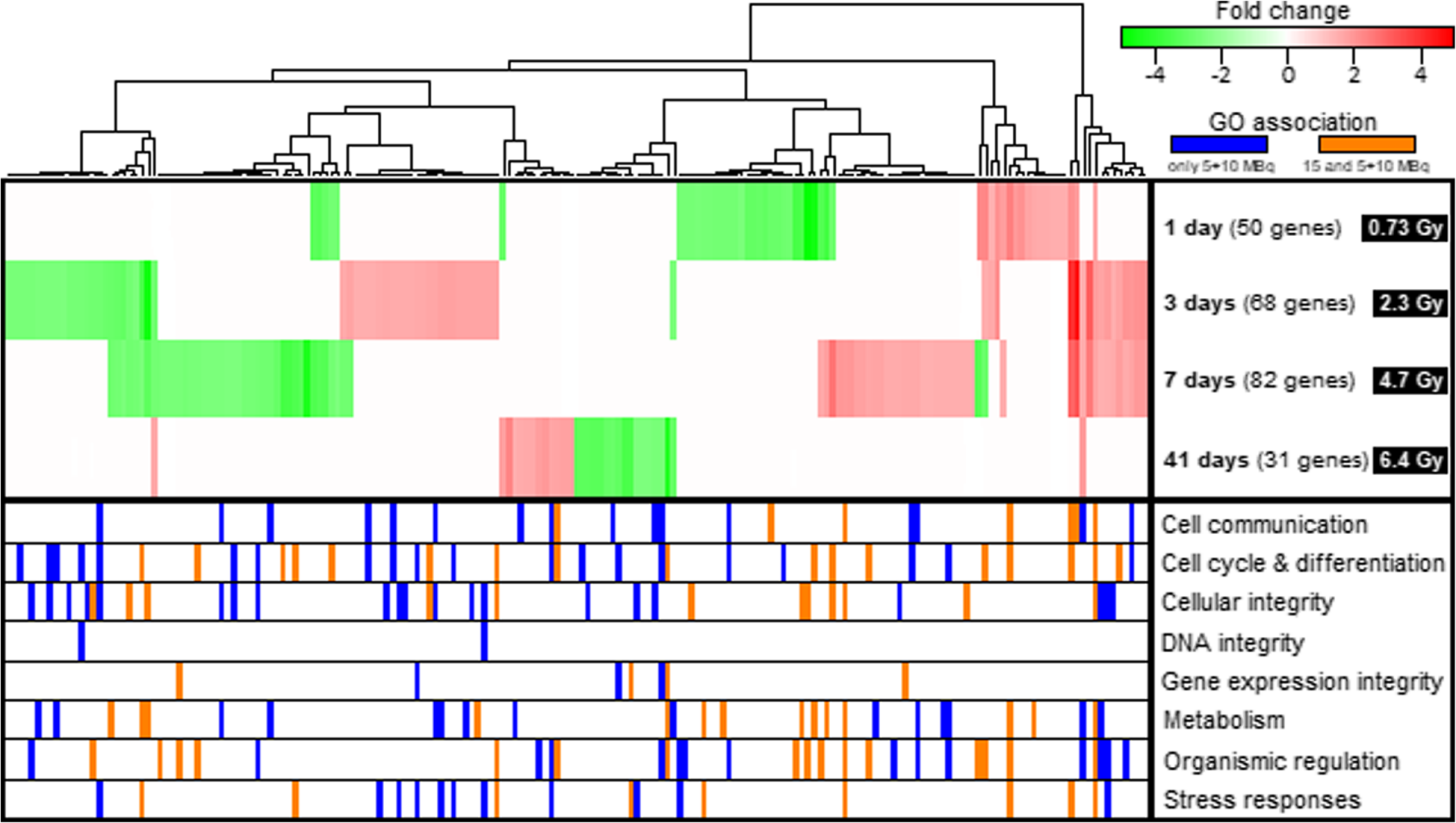
Distribution of significantly regulated genes after ^177^Lu-octreotate therapy with priming. Expression profiles of the 187 significantly regulated transcripts after i.v. injection of 5 + 10 MBq ^177^Lu-octreotate at 1, 3, 7, or 41 days after the last injection and annotation of enriched GO terms. Unsupervised hierarchical clustering was performed based on expression profiles. The mean absorbed dose to tumors processed for transcriptional analysis is indicated by the corresponding time point. Red and green indicate up- and downregulated transcripts (treated versus control, fold change ≥ 1.5, FDR-adjusted *p* < 0.01), respectively. In the lower segment, orange and blue indicate significant annotation (modified Fisher’s exact test, *p* < 0.05) of a gene to a GO term in a specified category. Orange indicates the GO annotation was significant both in animals treated with 5 + 10 MBq and in animals treated with 15 MBq single administration of ^177^Lu-octreotate (from GEO accession GSE80024), while blue indicates a significant GO annotation only in animals receiving 5 + 10 MBq ^177^Lu-octreotate

**Fig. 3:**
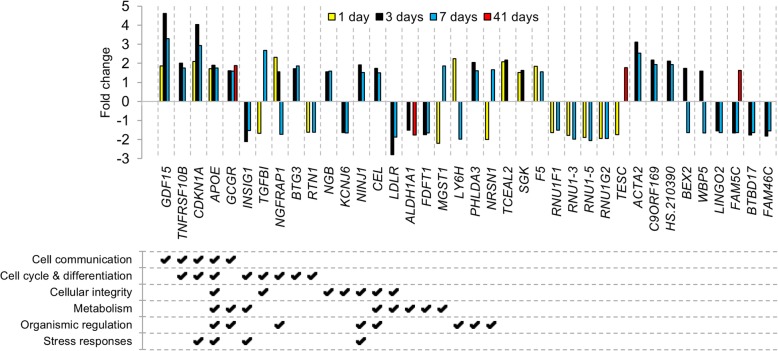
Expression profiles of commonly regulated transcripts after ^177^Lu-octreotate therapy with priming. Differential expression (treated vs. control) of the 38 significantly regulated transcripts shared between at least two of the studied time points, after i.v. injection of 5 + 10 MBq ^177^Lu-octreotate at 1, 3, 7, or 41 days after injection. Transcripts with fold change ≥ 1.5 and FDR-adjusted *p* < 0.01 were considered significantly regulated; annotation of enriched Gene Ontology terms was performed using the Gene Ontology database (modified Fisher’s exact test, threshold *p* < 0.05). Up- and downregulation is indicated by positive and negative values, respectively

A significant effect on gene expression levels was observed in GOT1 tumors after ^177^Lu-octreotate administration at all time points studied. In total, 187 differentially expressed genes were identified (microarray data was validated using the qPCR assay (Additional file 1: Table S1 and Additional file 2: Figure S1)). The number of regulated transcripts varied with time after injection (*n* = 31–82; Fig. 2). Of the detected transcripts, 33 (66%), 41 (60%), 48 (59%), and 27 (87%) were uniquely regulated at 1, 3, 7 and 41 days, respectively. Thirty-eight regulated transcripts were shared between at least two of the four time points (Fig. 3). Hierarchical clustering of the transcriptional profiles revealed similarities and differences in gene expression over time (Fig. 2). Notably, several of the transcripts associated with *Stress responses* were significantly regulated at 3 and 7 days, while transcripts with a pivotal role in maintaining DNA integrity were only significantly regulated at 3 days after the last injection.

Comparing the functional annotation to GO terms with the results seen after 15 MBq ^177^Lu-octreotate monotherapy (from GEO accession GSE80024), 43% of significant annotations were shared overall between the two treatment regimens (Fig. 2). Categorization of these annotated biological processes revealed that a majority of transcripts associated with *gene expression integrity* (57%) and *organismic regulation* (52%) were found in both 5 + 10 MBq and 15 MBq experiments. For the remaining six categories, most annotations were unique for the 5 + 10 MBq setting (48, 46, 45, 36, 30 and 0% shared annotations for the categories *Metabolism*, *Cell cycle & differentiation*, *Stress responses*, *Cellular integrity*, *Cell communication*, and *DNA integrity*, respectively). Furthermore, most of the annotations shared between the different regimens occurred at 1 and 7 days (90 and 58% shared annotations, respectively) after the last injection of ^177^Lu-octreotate, while 3 and 41 days showed more unique annotations (29 and 19% shared annotations, respectively).

### Differential effects on tumor cell proliferation and apoptosis in GOT1 tumors after ^177^Lu-octreotate therapy with priming

Analysis of affected biological functions using IPA predicted that a variety of functions related to tumor cell proliferation were significantly regulated at early time points after the last injection of ^177^Lu-octreotate (1 and 3 days), due to the regulation of, e.g., the *CDKN1A (p21)*, *GDF15*, and *SGK* genes (Table 1). Apoptotic processes were activated at 3 days after the last injection (*z* score = 2.0, *p* = 2.2 × 10^−5^), due to the regulation patterns of, e.g., the *BAX*, *GADD45A*, and *TNFRS10B* genes. Biological functions affected at 7 days were mainly related to cell migration, while a broader variety of functions (e.g., tumor sphere formation, proliferation of cancer cells, and budding of mitochondria) were affected during regrowth (41 days), due to the regulation of, e.g., *SOX2*, *CXCR7*, and *LGALS1*.

**Table 1:** Predicted biological functions affected in GOT1 tumors after ^177^Lu-octreotate therapy with priming

	Affected function	*z*	*p*	Targets from transcriptional data
1 day				
►	Hyperpolarization	–	1.7 × 10^− 4^	*↑SGK*, *↓SCN9A*
	G2 phase arrest in cancer	–	3.3 × 10^− 4^	*↑SGK*, *↑CDKN1A*
	Metabolism of D-glucose	–	1.6 × 10^− 3^	*↑APOE*, *↑APOD*
	Cell migration	–	2.2 × 10^−3^	*↑APOE*, *↑CDKN1A*
	Cancer cell morphology	–	2.2 × 10^−3^	*↑CDKN1A*, *↑GDF15*
3 days				
	G1 phase	1.0	1.1 × 10^−6^	*↑CEL, ↑APOE, ↑CCND3, ↑DDIT3, ↑GADD45A, ↑CDKN1A, ↑GDF15, ↓CDCA5, ↑BAX*
	Tumor cell proliferation	− 1.5	1.4 × 10^−5^	*↑BEX2*, *↓DLGAP5*, *↓CTGF*, *↑DDIT3*, *↑SGK*, *↑TNFRSF10B*, *↑GDF15*, *↑DDB2*, *↑BAX*, *↓PBK*, *↑VCAN*, *↓PARVB*, *↓FDFT1*, *↑CEL*, *↓ALDH1A1*, *↑CCND3*, *↑GADD45A*, *↑CDKN1A*, *↓CDCA5*
	Cell death in cancer	− 0.20	1.4 × 10^−5^	*↑DDIT3*, *↑GADD45A*, *↑SGK*, *↑BTG3*, *↑TNFRSF10B*, *↑CDKN1A*, *↑GDF15*, *↑BAX*
	Apoptosis in cancer	2.0	2.2 × 10^−5^	*↑CCND3*, *↑DDIT3*, *↑GADD45A*, *↑SGK*, *↑TNFRSF10B*, *↑CDKN1A*, *↑BAX*, *↓PBK*
	Cancer cell viability	− 1.5	2.7 × 10^−5^	*↑BEX2*, *↓CTGF*, *↓INSIG1*, *↑CDKN1A*, *↑GDF15*, *↓PBK*
7 days				
	Clustering of cancer cells	–	1.5 × 10^−5^	*↑CDH1*, *↑CDKN1A*
	Metabolism of cholesterol	–	2.8 × 10^−5^	*↑CEL*, *↑APOE*, *↓LDLR*, *↑ABCA1*
	Invasion of tumor	–	8.2 × 10^−5^	*↑APOE*, *↑CDH1*, *↑CTSL*, *↑GDF15*
	Quantity of intercellular junctions	–	9.0 × 10^−5^	*↑CDH1*, *↑GDF15*
	Invasion of cells	0.46	1.7 × 10^−4^	*↑KISS1R*, *↑CDH1*, *↑CTSL*, *RHOB*, *↑ACTA2*, *↑TGFBI*, *↑CDKN1A*, *↑GDF15*, *↓ENPP2*, *↓AGR2*, *↓BRINP3*, *↑SERPINE2*
41 days				
►	Quantity of Ca2+	–	2.2 × 10^−4^	*↑IAPP*, *↑GCGR*, *↑CCK*, *↓LGALS1*
►	Tumor sphere formation	–	6.0 × 10^−4^	*↓SOX2*, *↓CXCR7*
	Proliferation of cancer cells	− 1.2	1.3 × 10^−3^	*↓SOX2*, *↓ALDH1A1*, *↓CXCR7*, *↑PPP2R2C*
►	Apoptosis of T lymphoblasts	–	1.3 × 10^− 3^	*↓LGALS1*
►	Budding of mitochondria	–	1.3 × 10^−3^	*↓LGALS1*

Significantly affected biological functions identified with IPA (Fisher’s exact test, *p* < 0.05), ranked according to the lowest *p* value, for each time point. *z* scores indicate activation state of biological function; *z* > 2 indicates activation, and *z* < − 2 indicates inhibition. Up and down arrows indicate upregulated and downregulated genes in tumor samples from treated animals compared with controls, respectively. ► indicates the function was not affected in animals treated with 15 MBq single administration of ^177^Lu-octreotate (from GEO accession GSE80024)

Pathway analysis using IPA revealed a variety of significantly affected canonical signaling pathways (*p* < 0.05, Table 2). Several of the detected pathways are known to be involved in cancer development (e.g., PI3K/AKT signaling at 1 day, p53 signaling at 3 and 7 days, and Wnt/β-catenin signaling at 41 days) [25]. p53 (regulator of, e.g., DNA damage response) was also identified as an activated upstream regulator at early time points after the last injection of ^177^Lu-octreotate (*z* scores 3.3 and 1.9, *p* values 6.0 × 10^−14^ and 1.0 × 10^−6^, at 3 and 7 days, respectively, Table 3). Other upstream regulators with predicted activation states (|*z*| > 2) were ANXA2 (annexin A2, involved in the regulation of cellular growth and in signal transduction) and KDM5B (lysine demethylase 5B, a histone demethylase involved in the transcriptional repression of certain tumor suppressor genes) at 3 days (*z* scores − 2.0 and 2.6, *p* values 8.1 × 10^−9^ and 2.0 × 10^−5^, respectively) and PARP1 (poly (ADP-ribose) polymerase 1, involved in DNA strand break repair) at 7 days (*z* score − 2.0, *p* value 3.7 × 10^−7^) after the last injection of ^177^Lu-octreotate.

**Table 2:** Predicted canonical pathways affected in GOT1 tumors after ^177^Lu-octreotate therapy with priming

	Ingenuity Canonical Pathways	*p*	Targets from transcriptional data
1 day			
	Systemic lupus erythematosus signaling	1.3 × 10^−4^	*↓RNU1-3*, *↓RNU1-5*, *↓RNU1A3*
►	PI3K/AKT signaling	3.4 × 10^−3^	*↑PPP2R2B*, *↑CDKN1A*, *↑GDF15*
	Taurine biosynthesis	5.0 × 10^−3^	*↓CDO1*
►	Role of CHK proteins in cell cycle checkpoint control	8.3 × 10^−3^	*↑PPP2R2B*, *↑CDKN1A*
	L-cysteine degradation	1.0 × 10^−2^	*↓CDO1*
3 days			
	p53 signaling	3.2 × 10^−5^	*↑GADD45A*, *↑TNFRSF10B*, *↑CDKN1A*, *↑TIGAR*, *↑BAX*
	GADD45 signaling	3.2 × 10^−5^	*↑CCND3*, *↑GADD45A*, *↑CDKN1A*
►	Unfolded protein response	7.1 × 10^−4^	*↑DDIT3*, *↓INSIG1*, *↑HSPH1*
	Cholesterol biosynthesis	8.1 × 10^−4^	*↓FDFT1*, *↓MSMO1*
►	Death receptor signaling	3.6 × 10^−2^	*↑ACTA2*, *↑TNFRSF10B*
7 days			
	Systemic lupus erythematosus signaling	1.3 × 10^−4^	*↓RNU1-3*, *↓RNU1-5*, *↓RNU4-2*, *↓RNU4-1*
	LXR/RXR activation	1.3 × 10^−3^	*↓FDFT1*, *↑APOE*, *↓LDLR*, *↑ABCA1*
►	Epoxysqualene biosynthesis	7.8 × 10^−3^	*↓FDFT1*
►	p53 signaling	9.3 × 10^−3^	*↑TNFRSF10B*, *↑CDKN1A*, *↑SERPINE2*
►	Serotonin and melatonin biosynthesis	2.0 × 10^−2^	*↑TPH1*
41 days			
►	Role of Oct4 in mammalian embryonic stem cell pluripotency	1.6 × 10^−3^	*↓SOX2*, *↓NR2F2*
►	Lactose degradation	5.3 × 10^−3^	*↑GBA3*
►	CDK5 signaling	7.1 × 10^−3^	*↓EGR1*, *↑PPP2R2C*
	Embryonic stem cell differentiation into cardiac lineages	1.3 × 10^−2^	*↓SOX2*
►	Wnt/β-catenin signaling	2.0 × 10^−2^	*↓SOX2*, *↑PPP2R2C*

Significantly affected canonical pathways identified with IPA (Fisher’s exact test, *p* < 0.05), ranked according to the lowest *p* value, for each time point. Up and down arrows indicate upregulated and downregulated genes in tumor samples from treated animals compared with controls, respectively. ► indicates the pathway was not affected in animals treated with 15 MBq single administration of ^177^Lu-octreotate (from GEO accession GSE80024)

**Table 3:** Predicted upstream regulators affected in GOT1 tumors after ^177^Lu-octreotate therapy with priming

	Upstream regulator	*z*	*p*	Targets from transcriptional data
1 day				
	GDF15	–	6.1 × 10^−6^	*↑CDKN1A*, *↑GDF15*
	PPP5C	–	1.8 × 10^−5^	*↑CDKN1A*, *↑SGK*
	SATB1	0.11	7.8 × 10^−5^	*↑CDKN1A*, *↑F5*, *↑FAM129A*, *↑SGK*
	RNF2	–	1.3 × 10^−4^	*↑CDKN1A*, *↑GDF15*
	FH	–	2.2 × 10^−4^	*↑APOD*, *↑CDKN1A*
3 days				
►	p53	3.3	6.0 × 10^−14^	*↑ACTA2*, *↑APOE*, *↑BAX*, *↑CCND3*, *↑CDKN1A*, *↓CTGF*, *↑DDB2*, *↑DDIT3*, *↓FDFT1*, *↑GADD45A*, *↑GDF15*, *↑NINJ1*, *↓PBK*, *↑PHLDA3*, *↑SPATA18*, *↑TIGAR*, *↑TNFRSF10B*, *↑VCAN*
►	ANXA2	−2.0	8.1 × 10^−9^	*↑BAX*, *↑CDKN1A*, *↑GADD45A*, *↑TNFRSF10B*, *↑ZMAT3*
	MYC	−0.32	2.0 × 10^−8^	*↑BAX*, *↑CCND3*, *↑CDKN1A*, *↑DDB2*, *↑DDIT3*, *↓EXOSC8*, *↑GADD45A*, *↑HSPH1*, *↑TNFRSF10B*
	PPARGC1A	0.32	8.3 × 10^−8^	*↑BAX*, *↑CDKN1A*, *↓INSIG1*, *↓LDLR*, *↑TIGAR*
►	KDM5B	2.6	2.0 × 10^−5^	*↑DDIT3*, *↓DLGAP5*, *↑GADD45A*, *↓INSIG1*, *↓PBK*
7 days				
	PPARG	0.17	9.8 × 10^−8^	*↑ACTA2*, *↑CDH1*, *↑CDKN1A*, *↑CTSL*, *↓INSIG1*, *↓PDK4*
►	PARP1	−2.0	3.7 × 10^−7^	*↑CDH1*, *↓PEG10*, *↓TMSB15A*, *↑TNFRSF10B*
	p53	1.9	1.0 × 10^−6^	*↑ACTA2*, *↑APOE*, *↑CDH1*, *↑CDKN1A*, *↑F5*, *↓FDFT1*, *↑GDF15*, *↑NINJ1*, *↓PEG10*, *↑PHLDA3*, *↓TMSB15A*, *↑TNFRSF10B*
	SKI	–	1.1 × 10^−6^	*↑ACTA2*, *↑CDH1*, *↑CDKN1A*
	GDF15	–	1.5 × 10^−5^	*↑CDKN1A*, *↑GDF15*
41 days				
►	LIN28B	–	4.5 × 10^−5^	*↓BCL11A*, *↓SOX2*
	ID1	–	1.9 × 10^−4^	*↓EGR1*, *↓SOX2*
►	CDX2	–	1.1 × 10^−3^	*↓NR2F2*, *↓SOX2*
	SHP	–	1.3 × 10^−3^	*↓EGR1*
	mir-140	–	1.3 × 10^−3^	*↓SOX2*

Significantly affected upstream regulators identified with IPA (Fisher’s exact test, *p* < 0.05), ranked according to the lowest *p* value, for each time point. *z* scores indicate activation state of the upstream regulator; z > 2 indicates activation, and z < −2 indicates inhibition. Up and down arrows indicate upregulated and downregulated genes in tumor samples from treated animals compared with controls, respectively. ► indicates the upstream regulator was not affected in animals treated with 15 MBq single administration of ^177^Lu-octreotate (from GEO accession GSE80024)

## Discussion

The use of priming followed by a second administration of ^177^Lu-octreotate is a promising method to increase the efficacy of ^177^Lu-octreotate therapy of SSTR-expressing tumors. In the present study, gene expression profiling was used to study the mechanisms involved in the anti-tumor effect observed after treatment with ^177^Lu-octreotate including priming [12].

The anti-tumor effects of ^177^Lu-octreotate with different priming and second administration protocols have been presented in detail by Dalmo et al. [12]. The group of animals used in the present investigation showed tumor volume regression followed by tumor regrowth, i.e., a suboptimal treatment, chosen in order to be able to study also the regrowth period. Tumor mean absorbed doses were estimated to 6.4 Gy at infinity time for the 5 + 10 MBq administrations. This should be compared with the absorbed dose of 4.0 Gy to tumors in mice treated with 15 MBq single administration. Furthermore, statistically significant differences were observed in the tumor activity concentration between mice treated with and without priming therapy [12].

^177^Lu decays by beta emission but also has a gamma component [4]. The majority of the absorbed dose is delivered by the beta-particle, and although the gamma radiation has longer range, the photon contribution only marginally influences the absorbed dose due to the low yield of the emitted photons [5]. Even though this means the cross-absorbed fraction (dose delivered from, e.g., tumor to surrounding healthy tissues) is negligible, adverse effects in healthy tissues are still an issue due to the uptake of ^177^Lu-octreotate in healthy organs. The main dose-limiting organ for ^177^Lu-octreotate treatment are the kidneys, which accumulate the radiopharmaceutical partly due to SSTR expression but also because of reabsorption in proximal tubular cells [26]. While outside the scope of this work, the effects of ^177^Lu-octreotate on the kidney function and gene expression are important considerations and have been studied extensively by both us and others [19, 27–32].

A comparison of differentially regulated transcripts revealed significant differences across time points and indicated that different cellular functions are affected depending on the time after administration of ^177^Lu-octreotate. Approximately 60% of the transcripts differentially regulated at 1, 3, and 7 days were uniquely regulated at each time point, and at 41 days, the value was even higher with 87%. The microarray analysis revealed two response stages along the investigated time course, with a similarity between tumor responses at early time points (up to 7 days) compared with the response during tumor regrowth (41 days). This pattern is also illustrated by the 38 regulated transcripts shared between at least two of the time points studied, of which only four were found in the 41 days group. It is interesting to notice that the direction of regulation changed between early and late time points for *TESC* (tescalcin) and *FAM5C* (bone morphogenetic protein/retinoic acid-inducible neural-specific 3). Furthermore, a directional change was also found between day 1 and day 7 for *TGFB1*, *NGFRAP1* (involved in the extrinsic apoptotic signaling pathway [33]), *MGST1*, *LY6H* (involved in tissue morphogenesis), and *NRSN1*. TGFβ is an oncostatic regulator which, if mutated, is central in tumor cell proliferation, angiogenesis, and invasiveness. In NET, inactivation of this pathway has been reported in some cell lines, e.g., KRJ-I, but not in others, e.g., BON [34, 35]. The regulation of *TGFB1* in the present study is in coherence with results seen after 15 MBq ^177^Lu-octreotate monotherapy of GOT1 tumors [20] and may suggest functioning TGFβ-signaling in GOT1 tumors, but this finding remains to be proven.

IPA analysis of the data from 1 day after the last injection predicts that the initial response to treatment is growth arrest, based on, e.g., upregulation of the *CDKN1A* and *SGK* genes. Effects on tumor cell proliferation were also seen at 3 days after the last injection, along with an activation of apoptosis. This is in accordance with results seen after injection of 15 MBq ^177^Lu-octreotate [20]. However, in the present study on priming schedule, the target genes for the prediction of apoptosis activation suggest that both the intrinsic (via, e.g., the *BAX*, *GADD45A*, and *PBK* genes [36–38]) and extrinsic (via, e.g., the *TNFRSF10B* and *NGFRAP1* genes [33, 39]) apoptotic pathways are involved in the response. This is in contrast to the observed effects of 15 MBq monotherapy where only the intrinsic apoptotic pathway was affected [20]. In comparison with the results from the 15 MBq monotherapy study, no anti-apoptotic functions were affected during regrowth in the present study, and the downregulation of, e.g., *CXCR7* and *LGALS1* suggests an inhibition of cell proliferation. This may account for the slower regrowth observed with the priming treatment schedule.

In order to identify alterations in key regulatory pathways after ^177^Lu-octreotate therapy, analysis of IPA canonical pathways and upstream regulators was performed. Both the pathway and upstream regulator analysis revealed an effect on p53-signaling at 3 and 7 days after injection, with a predicted activation at 3 days (*z* score 3.3). Previous studies have shown that radiation exposure resulted in the activation of the p53 signaling pathway which, depending on the extent of DNA damage, promotes cell survival (by cell cycle arrest and DNA damage repair), or intrinsically activates cell death mechanisms such as apoptosis [40–42]. The predicted inhibition and activation of upstream regulators ANXA2 and KDM5B, respectively, to target genes such as *BAX*, *CDKN1A*, *GADD45A*, and *PBK* further suggests that tumor growth is suppressed via p53-mediated processes [43]. An effect on p53-signaling was also seen in GOT1 tumors in response to 15 MBq ^177^Lu-octreotate monotherapy, albeit only at 3 days after injection (compared with 3 and 7 days following treatment with priming) [20]. However, in the present study, PI3K/AKT signaling was also affected at 1 day, suggesting an increased effect on cell cycle arrest via upregulation of *PPP2R2B*, *CDKN1A*, *and GDF15*. The effect on the extrinsic apoptotic pathway (death receptor signaling) was also observed in the pathway analysis at 3 days, owing to the regulation of the *ACTA2* and *TNSRF10B* genes. Unfolded protein response (UPR) was also affected at 3 days. UPR is a stress response pathway which is caused by endoplasmic reticulum stress. Protein folding occurring in the endoplasmic reticulum is extremely sensitive to environmental changes regarding, e.g., reactive oxygen species (which could be caused by, e.g., ^177^Lu-octreotate-induced radiolysis of water or downstream effects of irradiation-induced cellular damage), hypoxia, or inflammatory stimuli, and studies have shown that endoplasmic stress can induce apoptosis (mediated by, e.g., JNK signaling) and enhances the radiosensitivity of tumor cells by degradation of RAD51 and subsequent reduction of double-strand break repair [44, 45]. Furthermore, the prediction of PARP1 as an inhibited upstream regulator at 7 days also suggests an impaired ability to repair DNA double-strand breaks. These responses were not seen in the study of 15 MBq ^177^Lu-octreotate monotherapy and may be contributing factors in the increased anti-tumor effect of a priming treatment schedule. Interestingly, we have previously demonstrated that the NAMPT inhibitor GMX1778 enhances the effects of single injection of 7.5 MBq ^177^Lu-octreotate treatment and induces a prolonged antitumor response in the same animal model as in the present study [46], an effect that may be related to PARP1 activation status.

Generation of GOT1 xenografts in nude mice is performed by first establishing a tumor into a few mice, by subcutaneous injection of cells from in vitro culture. After 4–6 months, the mice develop tumors at the site of injection. Tumors are then allowed to grow for several months and are then divided into 1-mm tissue pieces and transplanted into a larger number of mice, usually ca 60–100 each time. The tumor take is relatively low, tumors appear at different time points, the tumors grow slowly, and the growth rate differs much between animals within a transplantation batch, which results in a large variation in tumor sizes at a certain time point. Unfortunately, this sometimes results in a low number of tumor-bearing animals being available for experiments at a certain time, which limits the number of animals per treatment group. However, to our knowledge, the GOT1 model is unique in having a traceable neuroendocrine origin (a liver metastasis from a well-differentiated, serotonin-producing (enterochromaffin cell type) ileal NET) as well as harboring no mutations in p53 (mutated/dysfunctional p53 is usually not observed in patients) [47]. We therefore consider GOT1 to be the most representative model for studying small intestine NETs outside of using patient samples. Furthermore, the multifaceted differences in gene expression seen between treated and untreated groups in this work despite the strict statistical thresholding (fold change > 1.5, FDR-adjusted *p* < 0.01) suggests that gene regulation can be seen also with these group sizes.

## Conclusions

Microarray analysis characterized two stages of pathway regulation for the priming schedule (up to 1 week and around 1 month) which differed distinctly from cellular responses observed after monotherapy.

The priming treatment schedule resulted in induction of p53-mediated cell cycle arrest and apoptosis as well as extrinsically-mediated apoptosis in GOT1 tumors. Together with effects on PI3K/AKT-signaling and unfolded protein response, these findings suggest increased cellular stress in the tumors after a priming treatment schedule compared with conventional ^177^Lu-octreotate monotherapy. Furthermore, downregulation of, e.g., *CXCR7* and *LGALS1* suggests an inhibition of cell proliferation at late time points after the last injection, which may explain the slow regrowth compared with tumors in animals treated with monotherapy.

## Additional files

Additional file 1: **Table S1.** qPCR validation of microarray data. (DOCX 14 kb)

Additional file 2: qPCR validation of microarray data. (TIF 933 kb)

## Abbreviations

BASE: BioArray software environment
GEO: Gene Expression Omnibus
GO: Gene Ontology
IPA: Ingenuity Pathway Analysis
MIRD: Medical internal radiation dose
NCBI: National Center for Biotechnology Information
NET: Neuroendocrine tumor
qRT-PCR: Quantitative reverse transcription-polymerase chain reaction
SCIBLU: Swegene Center for Integrative Biology
SSTR: Somatostatin receptor
UPR: Unfolded protein response
