# Supplementary material for: Transcriptional effects of 177Lu-octreotate therapy using a priming treatment schedule on GOT1 tumor in nude mice
Source: EJNMMI Res. 2019 Mar 20;9:28. doi: 10.1186/s13550-019-0500-2 (PMC6426909; doi:10.1186/s13550-019-0500-2)
Supplement: Supplementary file 1 — Table S1. qPCR validation of microarray data. (DOCX 14 kb) [file 13550_2019_500_MOESM1_ESM.docx]

| **Table A1:** qPCR validation of microarray data | | | | | | | | | | | | | |
| --- | --- | --- | --- | --- | --- | --- | --- | --- | --- | --- | --- | --- | --- |
|  |  |  | **1 day** | |  | **3 days** | |  | **7 days** | |  | **41 days** | |
| Target gene | TaqMan assay ID | Illumina probe ID | **qPCR** [log_2_-ratio] | **Microarray** [log_2_-ratio] |  | **qPCR** [log_2_-ratio] | **Microarray** [log_2_-ratio] |  | **qPCR** [log_2_-ratio] | **Microarray** [log_2_-ratio] |  | **qPCR** [log_2_-ratio] | **Microarray** [log_2_-ratio] |
| *BAX* | Hs99999001_m1 | 2321064 | -0.063 | *n.s.* |  | 0.96 | 0.81 |  | 0.25 | *n.s.* |  | 0.39 | *n.s.* |
| *CDKN1A* | Hs00355782_m1 | 1784602 | 0.81 | 1.1 |  | 2.7 | 2.0 |  | 1.5 | 1.6 |  | 0.71 | *n.s.* |
| *FDFT1* | Hs00926054_m1 | 1741096 | -0.81 | *n.s.* |  | -0.77 | -0.81 |  | -0.68 | -0.72 |  | -6.4 | *n.s.* |
| *GDF15* | Hs00171132_m1 | 2188862 | 1.0 | 0.90 |  | 3.5 | 2.2 |  | 3.2 | 1.7 |  | 0.73 | *n.s.* |
| *TGFBI* | Hs00932747_m1 | 1663866 | -2.5 | -0.75 |  | 0.11 | *n.s.* |  | 2.6 | 1.4 |  | 3.0 | *n.s.* |
| *ACTA2* | Hs00426835_g1 | 1671703 | 0.51 | *n.s.* |  | 3.0 | 1.6 |  | 2.4 | 1.3 |  | 0.37 | *n.s.* |
| *LY6H* | Hs01108584_m1 | 2237252 | 1.9 | 1.2 |  | 0.21 | *n.s.* |  | -1.3 | -0.98 |  | 0.64 | *n.s.* |
| *LDLR* | Hs01092524_m1 | 2053415 | -1.2 | *n.s.* |  | -2.9 | -1.5 |  | -1.8 | -0.91 |  | 0.66 | *n.s.* |
| *EGR1* | Hs00152928_m1 | 1762899 | 0.20 | *n.s.* |  | 0.61 | *n.s.* |  | 1.7 | *n.s.* |  | -0.66 | -1.1 |
| Reference genes were chosen from microarray data that showed high overall intensity and low overall transcriptional variation (*i.e. EEF1A1* (Taqman assay ID: Hs00265885_g1), *RPL6* (Taqman assay ID: Hs03044365_g1) and *RPS12* (Taqman assay ID: Hs00831630_g1)). *n.s.* indicates non-significant result from microarray data. | | | | | | | | | | | | | |
