# Supplementary figures and images for: Transcriptional effects of 177Lu-octreotate therapy using a priming treatment schedule on GOT1 tumor in nude mice
Source: EJNMMI Res. 2019 Mar 20;9:28. doi: 10.1186/s13550-019-0500-2 (PMC6426909; doi:10.1186/s13550-019-0500-2)

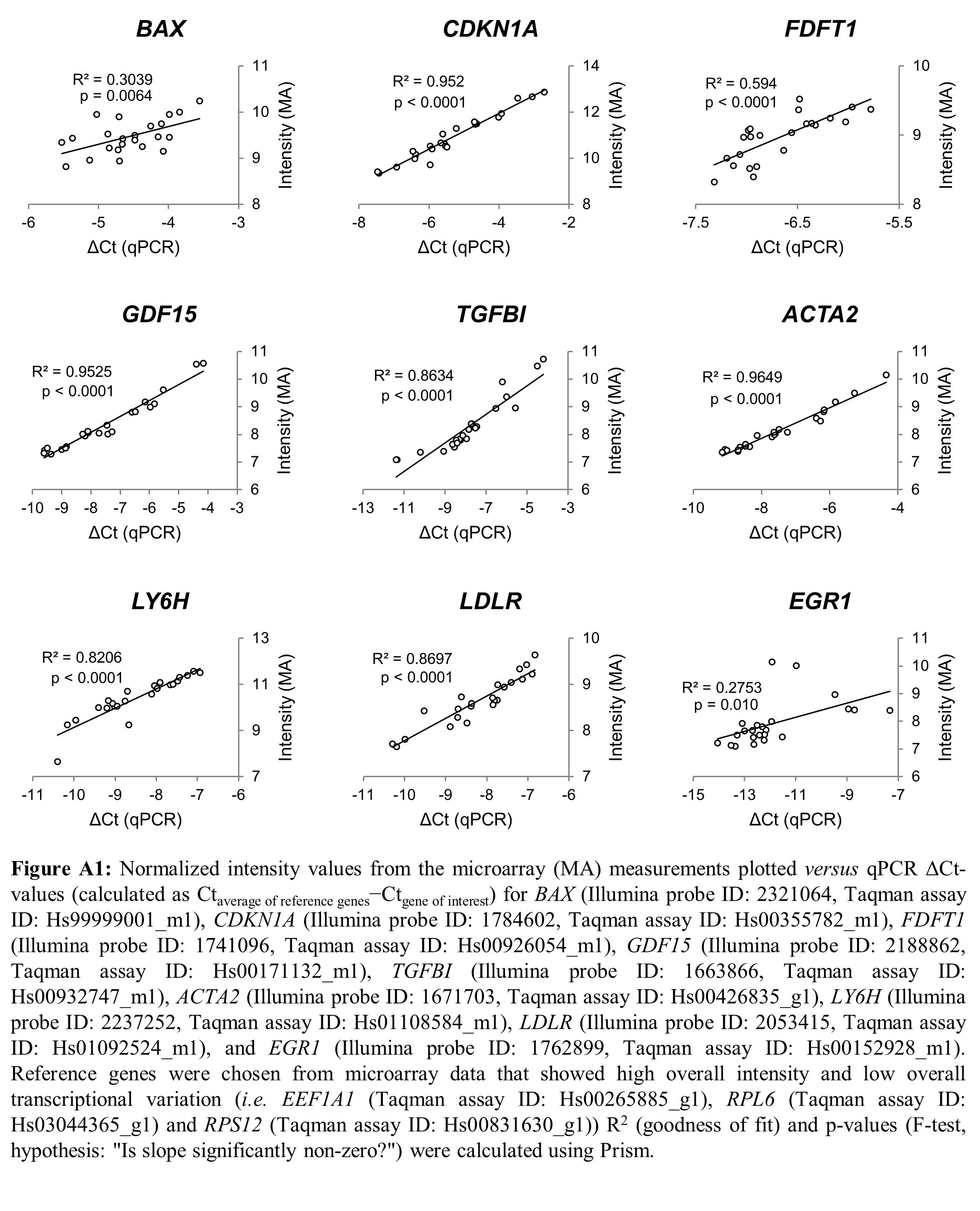

Supplement: Supplementary file 2 — qPCR validation of microarray data. (TIF 933 kb) [file 13550_2019_500_MOESM2_ESM.tif]
